# Machine learning-enabled UAV hyperspectral identification of tomato spotted wilt virus in tobacco

**DOI:** 10.3389/fpls.2025.1728043

**Published:** 2025-12-09

**Authors:** Chuntang Mao, Yanan Zhao, Leiguang Wang, Ziyi Yang, Weili Kou, Weiheng Xu, Huan Wang, Xiaolong Zhang, Ning Lu, Guangzhi Di

**Affiliations:** 1 College of Big Data and Intelligent Engineering, Southwest Forestry University, Kunming, Yunnan, China; 2 College of Landscape Architecture and Horticulture, Southwest Forestry University, Kunming, Yunnan, China; 3 Research and Development Center, Yunnan Wooja Biotechnology Co., Ltd, Kunming, Yunnan, China; 4 Office of the President, Southwest Forestry University, Kunming, Yunnan, China

**Keywords:** tobacco plants, tomato spotted wilt virus, UAV, hyperspectral imaging, machine learning

## Abstract

**Problems:**

Tomato Spotted Wilt Virus (TSWV) severely affects tobacco yield and quality, creating an urgent need for accurate, rapid, non-destructive monitoring to support disease management. While existing TSWV detection methods perform well at the leaf scale, their field-scale application remains challenging. Due to complex crop canopy structures, spectral characteristics at the field level differ significantly from leaf-level observations, and TSWV-sensitive spectral features are still unclear. This study therefore aims to develop a field-scale TSWV identification model using UAV-based hyperspectral imaging to enable targeted disease control.

**Methodology:**

A UAV-mounted hyperspectral camera (400–1000 nm) was deployed to capture imagery of tobacco plants at the rosette stage, enabling comparative spectral analysis between healthy and infected specimens. To identify sensitive features associated with tobacco plants infected with TSWV, six distinct feature extraction methodologies encompassing traditional statistical approaches (spectral ratio, correlation analysis, and principal component analysis [PCA]), machine learning-based techniques (relevant features [Relief], successive projections algorithm) and vegetation indices were utilized. Subsequently, we conducted a systematic evaluation of 18 classification models developed using three machine learning algorithms—support vector machine (SVM), k-nearest neighbors, and extreme gradient boosting —with the derived feature variables.

**Results:**

This study demonstrates that while all integrated models combining Relief- and Correlation- selected feature bands with three machine learning algorithms delivered excellent performance, the SVM-Relief model achieved the most outstanding results (OA = 97.3%, AUC = 0.994, Kappa=0.947). Based on the SVM-Relief combination, a proposed method called RPR —which integrates PCA with recursive feature elimination— was further employed to reduce the number of feature indicators from 15 to 4 (775.6/772.9/781.1/756.4 nm). The resulting SVM-RPR combination model achieved performance (OA = 97.3%, AUC = 0.990, Kappa=0.947) comparable to that of the SVM-Relief model.

**Contribution:**

This indicated that red-edge bands were of significant value in distinguishing healthy and TSWV-infected tobacco plants. Our study indicates the significant potential of integrating UAV-based hyperspectral imaging with machine learning techniques for rapid, non-destructive detection of tobacco TSWV at the field scale. The proposed approach offers a novel and efficient pathway for remote sensing-based monitoring of viral diseases in crops, with implications for precision agriculture and plant disease management.

## 1 Introduction

Tobacco is an important economic crop in China, and its quality and yield are easily affected by pests and diseases throughout the entire growing season. Tomato spotted wilt virus (TSWV), as one of the key diseases affecting the quality and yield of tobacco leaves, has been identified in multiple provinces throughout China, with particularly high prevalence in Yunnan Province, a major tobacco-producing region (Jiang et al., 2017). Thus, accurate, rapid, and non-destructive identification of TSWV is of great practical significance for formulating and implementing disease management strategies and guiding tobacco cultivation practices.

Tomato spot wilt (TSW), caused by TSWV, was first confirmed in flue-cured tobacco in Georgia in 1986. Tobacco plants are susceptible to the disease from the seedling stage through to maturity in the field. Infected plants exhibit stunted growth, with symptoms including drooping or bent top buds and twisted leaves, leading to asymmetric development. In severe cases, infected plants cease growth for weeks, display drooping leaves and eventually die, resulting in significant economic losses (Srinivasan et al., 2017). Previous studies have demonstrated that TSWV can be effectively managed through an integrated approach encompassing physical, biological, and chemical control strategies (Chatzivassiliou, 2008). However, the cornerstone of successful disease management lies in the rapid and accurate acquisition of disease occurrence status and the understanding of the disease incidence rate, so as to formulate and implement disease control measures.

Traditional approaches for monitoring crop diseases predominantly rely on field investigations conducted by professionals to collect relevant data (Fang and Ramasamy, 2015). However, these conventional methods are often associated with significant drawbacks, including time-consuming and labor costs, limited accuracy, delayed responsiveness, and susceptibility to human error. When addressing large-scale disease outbreaks, these limitations are further exacerbated by their inherent subjectivity and lack of timeliness, thereby hindering the implementation of effective prevention and control strategies. Consequently, the development of real-time and efficient monitoring systems to track the occurrence and progression of diseases, coupled with the timely application of control measures to minimize crop losses, represents a critical challenge in modern agricultural production. In this context, hyperspectral imaging technology offers a promising solution, as it enables the simultaneous acquisition of both spatial and spectral information, thereby providing a comprehensive and precise tool for disease detection and analysis.

Infection by plant diseases can induce significant alterations in the biophysical and biochemical properties of plants, including modifications in tissue structure, intercellular space, transpiration rate, pigment content, and water content (Slaton et al., 2001; Rumpf et al., 2010). These physiological and structural changes often manifest as variations in the spectral characteristics of plants, which can be effectively captured using hyperspectral imaging platforms (Zarco-Tejada et al., 2013). Extensive research has been conducted on the application of hyperspectral imaging for the identification of viral diseases in tobacco at the leaf scale. For instance. For instance, the Successful Projects Algorithm (SPA) was employed to extract characteristic spectral wavelengths at 554.07, 574.31, 760.16, 791.78, 839.55, and 936.33 nm at the leaf scale for identifying TSWV-infected tobacco leaves, achieving the highest accuracy of 85.2% when coupled with the Boosted Regression Trees (BRT) model (Gu et al., 2019) Similarly, hyperspectral reflectance data within the visible and near-infrared spectral ranges have been demonstrated to differentiate between TSWV-infected and healthy tobacco leaves (Zhu et al., 2017). By applying statistical analysis methods, they were able to detect the progression of TSWV infection in tobacco plants, identifying measurable differences between infected and healthy plants as early as 14 days post-infection. These studies underscore the potential of hyperspectral imaging as a powerful tool for early and accurate detection of plant diseases, particularly in the context of tobacco viral infections. However, current research efforts have predominantly focused on TSWV identification utilizing stationary hyperspectral imaging systems at the leaf scale under controlled conditions. While these methodologies enable the acquisition of sub-millimeter resolution spectral data, facilitating the precise detection of early disease symptoms including leaf vein discoloration and small disease spots (Abdulridha et al., 2020), the inherent limitations of single-leaf scanning approaches, particularly their labor-intensive and time-consuming procedures, coupled with their failure to effectively address field-scale challenges such as mixed pixels and complex canopy structure, render them impractical for large-scale field applications (Zhao et al., 2022).

Comparative analyses reveal that unmanned aerial vehicle (UAV)-based remote sensing platforms demonstrate superior operational capabilities relative to conventional leaf-scale monitoring approaches, particularly in terms of temporal efficiency (rapid data acquisition), spatial extensiveness, and economic viability (Zhao et al., 2024). The integration of hyperspectral imaging sensors with UAV systems facilitates the acquisition of high spatiotemporal resolution datasets, thereby providing unprecedented opportunities for landscape-level crop monitoring. Nevertheless, inherent technical constraints persist, as UAV-derived hyperspectral imagery is characterized by reduced spatial resolution compared to leaf-scale measurements, resulting in spectral mixing phenomena that obscure disease symptom signatures (Xue et al., 2021). This resolution discrepancy hinders the effective translation of leaf-scale spectral signatures to canopy-level monitoring approaches. Given these technical limitations and the escalating threat of TSWV to tobacco production systems, there exists a critical research imperative to develop and validate UAV-based hyperspectral protocols for field-scale disease diagnostics.

Hyperspectral sensors have exhibited considerable potential in detecting subtle spectral variations in crops induced by diseases (Berger et al., 2018). However, the high dimensionality and multicollinearity inherent in hyperspectral data acquired by unmanned aerial vehicles (UAVs) present significant challenges in identifying disease-sensitive spectral indicators, which are critical for reducing computational complexity and improving model efficiency. To address these challenges, researchers have widely adopted sensitive feature extraction methods based on statistical and machine learning algorithms. For instance, variance analysis was employed to identify sensitive features from hyperspectral data, achieving an accuracy of 81% in recognizing grape leaf curl disease using a discriminative classifier (Naidu et al., 2009). Similarly, correlation analysis was utilized to select sensitive spectral features from hyperspectral data, and kernel discriminant analysis was applied to distinguish wheat stripe rust, aphids, and powdery mildew, achieving a maximum accuracy of 89.2% (Shi et al., 2017). Furthermore, the SPA was leveraged to extract sensitive features for detecting blight diseases on tomato leaves, with an overall classification accuracy ranging from 97.1% to 100% (Xie et al., 2015). These studies highlight the effectiveness of statistical and machine learning-based methodologies in improving the accuracy and efficiency of disease detection through the utilization of hyperspectral data.

Although feature extraction significantly reduces the amount of hyperspectral data, utilizing these features to construct efficient and robust classification models remains the key to achieving accurate disease recognition (Heylen et al., 2014). To improve the prediction accuracy of the model, multiple machine learning (ML) algorithms are also applied to build recognition models for distinguishing between healthy and diseased plants, such as Support Vector Machine (SVM) (Were et al., 2015), Extreme Gradient Boosting (XGBoost) (Ge et al., 2021), K-Nearest Neighbor (KNN) (Cao et al., 2018), etc. The recognition performance of ML techniques in plant disease identification exhibits significant variability due to their differential sensitivity to structural characteristics of hyperspectral data, including spectral dimensionality, noise distribution patterns, and feature correlation coefficients (Duan et al., 2019). This phenomenon persists even when analyzing pathologically similar viral infections in different plants. For instance, an early detection model for TSWV-infected tobacco leaves was developed using Boosted Regression Trees (BRT), achieving an overall accuracy of 85.2% (Gu et al., 2019). In the early detection of TSWV in pepper leaves, an accuracy of 96.25% was achieved by employing an improved OR-AC-GAN (Mensah et al., 2024). Furthermore, a Back-Propagation Neural Network (BPNN) was demonstrated to achieve up to 95% accuracy in identifying early-stage Tobacco mosaic virus (TMV) infections (Zhu et al., 2017). These studies collectively indicate that integrating ML algorithms with hyperspectral imaging enables effective discrimination of virus-infected tobacco leaves by leveraging spectral signatures associated with physiological stress response. However, even for similar diseases, the optimal ML algorithms for identifying virus-infected tobacco leaves in the above studies are not the same. Meanwhile, these studies are all based on identification models constructed at the leaf scale, and there is uncertainty as to whether they can be applied to identify TSWV-infected tobacco plants at the field scale.

More specifically, while previous studies have explored leaf-scale identification techniques for TSWV, the applicability of leaf-scale spectral characteristics to field-scale monitoring remains uncertain. The structural heterogeneity of crop canopies alters spectral signatures compared to isolated leaf measurements, and definitive spectral biomarkers indicative of TSWV infection have yet to be conclusively identified. Furthermore, hyperspectral remote sensing using UAVs for TSWV detection in tobacco has not yet been systematically investigated. To address these research issues, this study focuses on field-scale hyperspectral identification of TSWV-infected tobacco plants using UAV-based remote sensing, aiming to develop an effective monitoring approach for TSWV. The objectives of this study were as follows:

1. To exploit optimal spectral indicators related to TSWV by comparing different feature selection methods.
2. To determine the optimal combination of feature selection methods and machine learning models for accurate detection of TSWV-infected tobacco plants.
3. To assess the feasibility of UAV-based hyperspectral detection for TSWV-infected tobacco plants at the field scale.

## 2 Materials and methods

### 2.1 Study area

**Figure 1:**
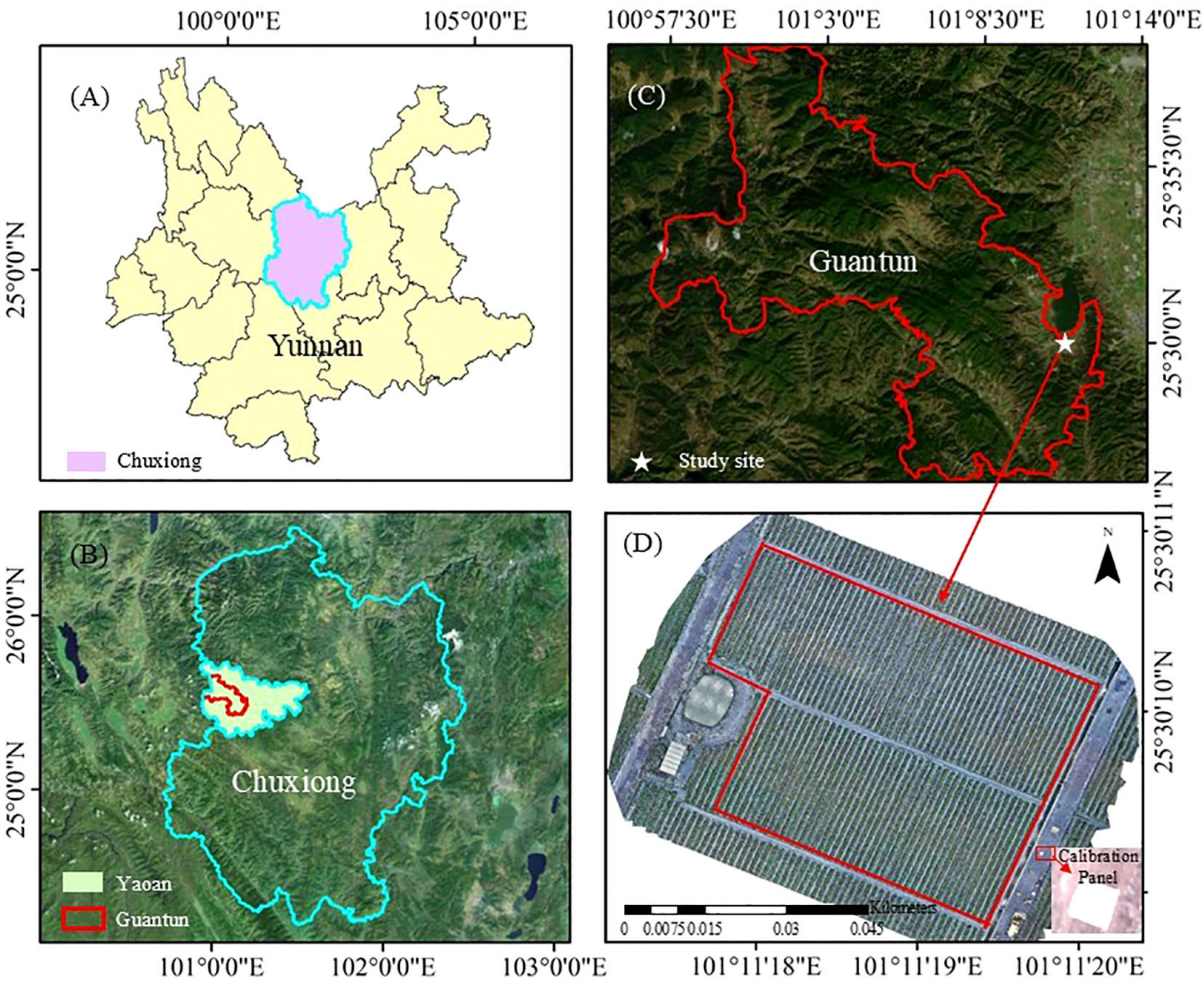
The location of the study area. **(A)** Yunnan Province. **(B)** Yao’an County, Chuxiong Yi Autonomous Prefecture. **(C)** Guantun Town. **(D)** Study area.

The field experiment was conducted in the core tobacco cultivation region of Guantun Town, Yao’an County, Chuxiong Yi Autonomous Prefecture, Yunnan Province, China (25°30′11″N,101°11′18″E) (**Figure 1**), characterized by a subtropical monsoon climate. The flue-cured tobacco variety MS Yunyan 87 was used in this experiment, with transplanting completed on April 28, 2024. The seedling method used was the floating seedling system. The experimental field was characterized by red soil, and fertilizer application was done in two stages to ensure optimal nutrient supply. On April 28, during the day of transplanting, a basal application of compound fertilizer (N: P_2_O_5_: K_2_O = 15: 15: 28) was administered at a rate of 600 kg/ha, where the fertilizer was applied in a ring pattern 5 cm away from the base of the tobacco plants and then covered with soil. This method ensured that the roots of the tobacco seedlings did not come into direct contact with the basal fertilizer while minimizing nutrient loss from premature application (Yaru et al., 2021). Subsequently, on May 20, a supplementary fertilization was performed by uniformly mixing 150 kg/ha of the same compound fertilizer with 150 kg/ha of nitrogen-potassium fertilizer (N: P_2_O_5_: K_2_O = 12.5: 0: 33.5), which was then applied as a 5% aqueous solution through drenching to enhance nutrient availability and uptake. The experimental plot covered an area of 0.25 hectares on relatively flat terrain. The tobacco plants were arranged at a spacing of 0.5 m between plants and 1.2 m between rows, with a total population of 3650 plants. According to the field investigation conducted on May 31, 2024, the incidence rate of TSWV exceeded 5%, which was representative of the local tobacco growth and disease prevalence conditions.

### 2.2 Data acquisition and preprocessing

#### 2.2.1 TSWV field investigation

**Figure 2:**
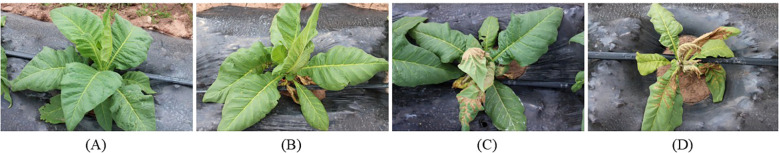
Representative images of tobacco plants at different TSWV severity grades. **(A)** Healthy. **(B)** Mild. **(C)** Moderate. **(D)** Severe.

Field investigations were conducted during the rosette stage of tobacco plants, a critical phenological phase that coincides with the epidemiological peak of TSWV infection. Due to the relatively short period after transplanting and effective field management, weed infestation was negligible. The ground cover primarily consisted of tobacco plants, plastic mulch, and bare soil, providing optimal conditions for disease monitoring. Disease diagnosis was established according to three definitive pathognomonic indicators of TSWV: (1) acute apical leaf distortion with ≥30° upward margin curling, (2) shoot apex deviation showing S-shaped curvature, (3) vascular necrosis and pith cavitation presenting as blackened veins. A team of plant pathologists specializing in TSWV performed systematic field assessments using a comprehensive manual assessment protocol to (1) identify TSWV symptomatic plants and (2) classify disease severity according to established grading criteria for each plant within the study area. To ensure diagnostic accuracy, symptom classification strictly adhered to the binding national standard GB/T 23222-2008 (“Tobacco Disease Severity Investigation and Classification”) (Gao et al., 2015). The detailed TSWV classification criteria and disease severity diagrams are presented in **Table 1**, **Figure 2**, respectively. Furthermore, to enable precise georeferencing and identification of TSWV-infected tobacco plants in UAV-acquired imagery, a real-time kinematic instrument called ZHD V200 (RTK, Guangzhou Hi-Target Navigation Tech Co., Ltd., China) was employed to acquire centimeter-level accuracy of diseased plant locations.

**Table 1:** Description of representative symptoms for different TSWV severity grades in tobacco plants.

TSWV grade	Symptom description	Affected area
Healthy	Asymptomatic and normally developed plant	0
Mild	Mild curling of 1–2 apical leaves without necrotic spots	<10%
Moderate	Systemic curling in >50% of leaves with scattered chlorotic spots	10% - 40%
Severe	Severely deformed leaves with >30% leaf area exhibiting brown necrosis, accompanied by plant stunting	>40%

#### 2.2.2 Image acquisition

Hyperspectral image acquisition was conducted on June 2, 2024, corresponding to the rosette stage of the tobacco plants. This growth stage was selected to avoid mutual shading effects on canopy structure, as the plants—with heights and canopy widths of 25–30 cm—exhibited negligible canopy overlap.

**Figure 3:**
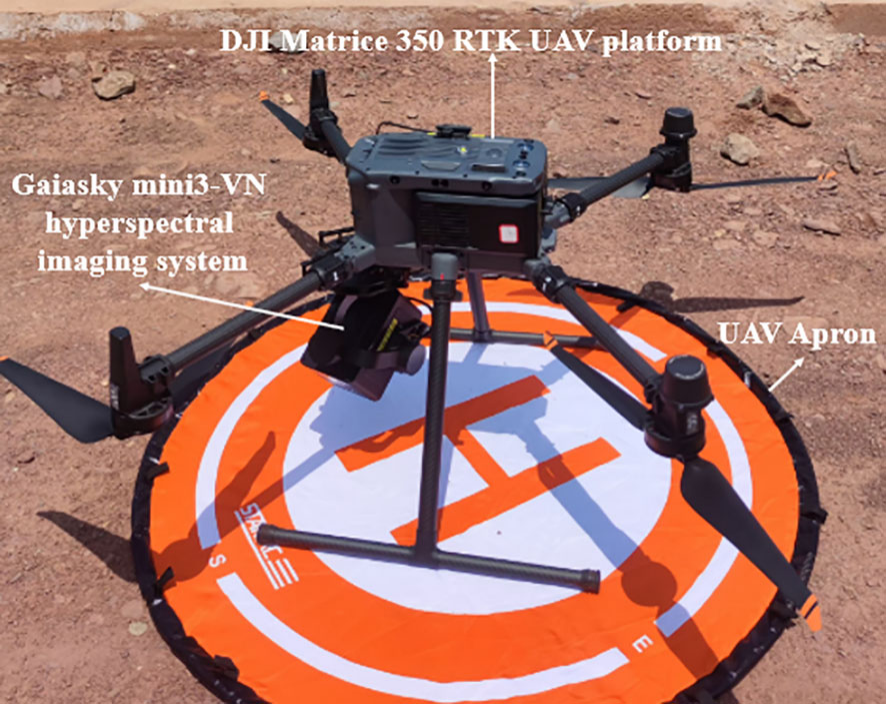
UAV-based hyperspectral imaging system.

To capture the subtle spectral variations characteristic of TSWV-infected tobacco plants, this investigation employed an advanced hyperspectral remote sensing system. The Gaiasky mini3-VN hyperspectral imaging system (Dualix Spectral Imaging, China), featuring a spectral range of 400–1000 nm, 2.7 nm spectral sampling interval, and 224 contiguous spectral bands, was integrated with a DJI Matrice 350 RTK UAV platform (SZ DJI Technology Co., Ltd., China) (**Figure 3**). The UAV platform, with a maximum flight endurance of 55 minutes and a 9 kg maximum takeoff weight, was equipped with a real-time kinematic positioning system to ensure precise geolocation of acquired data.

A comprehensive calibration protocol was implemented to ensure data quality and accuracy. Pre-flight calibration procedures included the acquisition of calibration panel images using the hyperspectral imaging system for spectral calibration. Furthermore, a 1.2 m × 1.2 m calibration panel with 50% reflectance was strategically positioned within the study area to facilitate the conversion of hyperspectral radiance measurements to reflectance values through empirical line calibration methods (Smith and Milton, 1999).

To optimize illumination conditions and reduce shadow effects, all data collection was scheduled between 11:00 and 14:00 local time, corresponding to periods of maximal solar elevation. The UAV was maintained at a constant altitude of 75 meters above ground level, yielding a ground sampling distance (GSD) of approximately 5 cm, which provided sufficient spatial resolution for detailed analysis at the individual plant level while ensuring adequate coverage of the study area.

#### 2.2.3 Image processing

To eliminate the influence of environmental factors on hyperspectral images during image acquisition and ensure the physical authenticity of spectral data, a systematic preprocessing workflow was applied. Reflectance products were first derived using SpecVIEW spectral imaging software (Dualix Spectral Imaging, Nanjing, China), incorporating three key steps: (1) Lens Correction– Addressing optical distortions; (2) B&W (Brightness & White) Correction – Normalizing illumination variability; (3) Atmospheric Correction – Compensating for aerosol and water vapor effects to generate reflectance products. Subsequently, HiRegistrator software (Dualix Spectral Imaging, Nanjing, China) performed geometric correction and orthorectification to generate high-precision orthomosaic images covering the entire experimental area.

**Figure 4:**
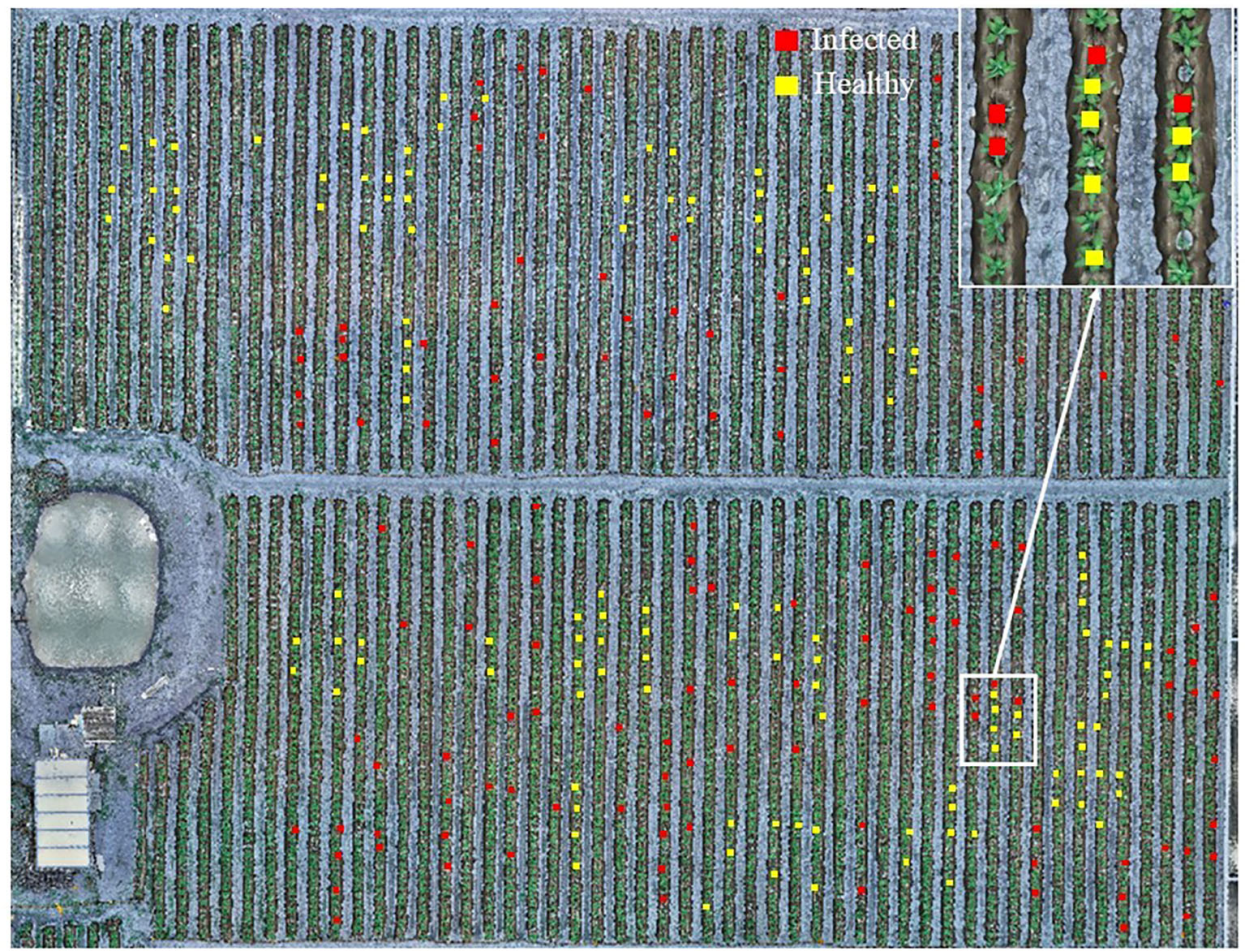
Hyperspectral images of the experimental field with UAV (The red and yellow rectangles represent the TSWV-infected and healthy tobacco plant samples in the field investigation, respectively).

**Figure 5:**
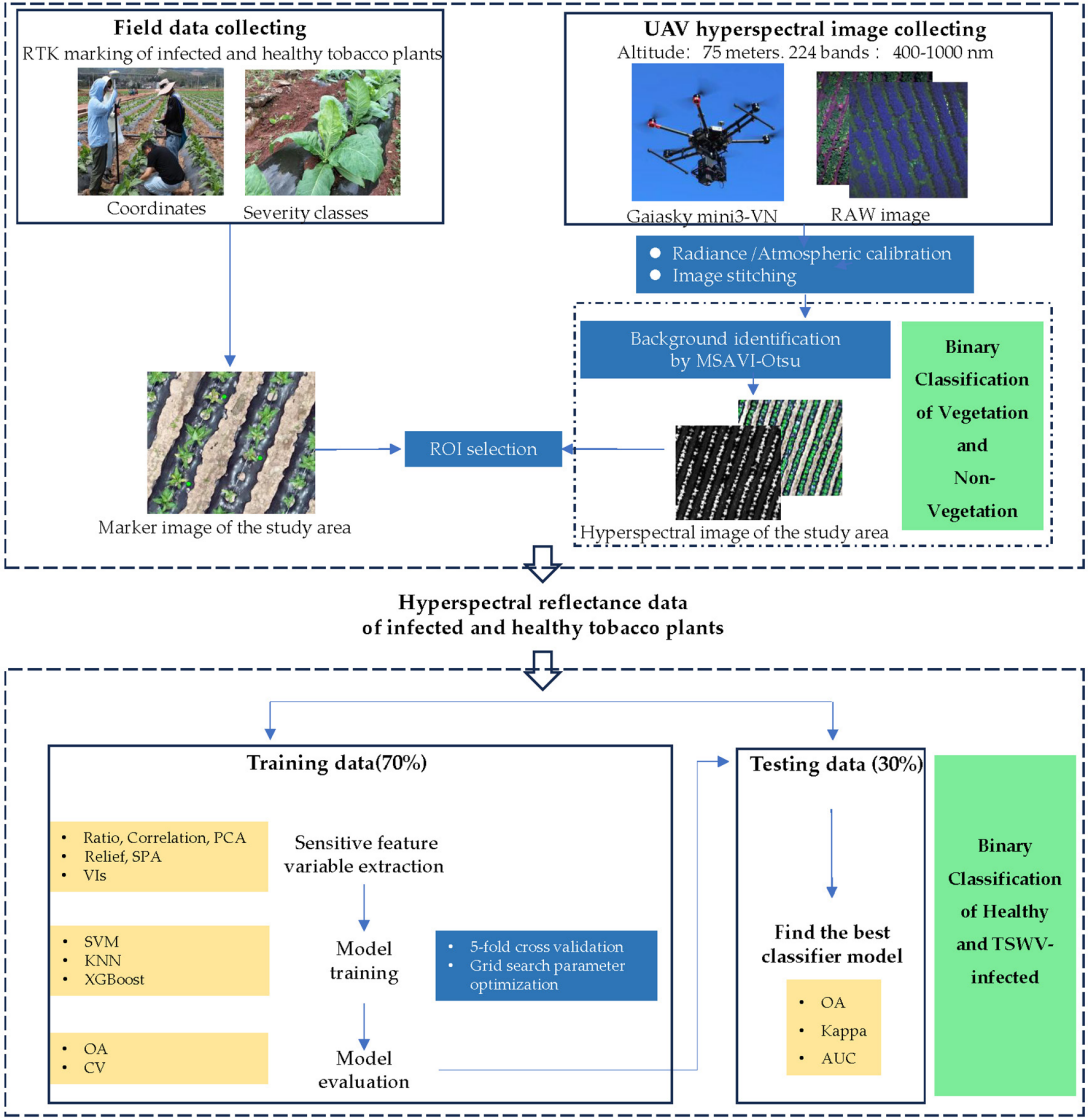
Flowchart of this study.

After obtaining the corrected and stitched hyperspectral images, the study implemented three-class classification (non-vegetation background/healthy plants/diseased plants) through a hierarchical two-stage approach. Stage 1: Non-vegetation background separation calculates the Modified Soil Adjusted Vegetation Index (MSAVI) across the entire orthomosaic to suppress bare soil and plastic mulch noise. A total of 48 samples from TSWV-infected tobacco plants, 48 samples from healthy plants, 30 soil samples, and 30 plastic mulch samples were analyzed. The Otsu method (Otsu, 1975) was employed to determine the adaptive optimal threshold for distinguishing between non-vegetation (labeled as pixel value 0, representing soil and plastic mulch as background) and vegetation (tobacco plants). This step enabled efficient classification of healthy and diseased tobacco plants in subsequent analyses. Stage 2: After background removal, rectangular ROIs (3×3 pixels) were manually centered on individual tobacco plants within orthomosaic images, leveraging RTK-derived coordinates for precise localization to obtain representative reflectance data of healthy and diseased tobacco plants. In this study, the mean reflectance values of each ROI were used to represent sample measurements. A total of 250 spectral reflectance samples were collected from field-marked tobacco plants, comprising 122 samples from TSWV-infected tobacco plants and 128 samples from healthy plants (**Figure 4**). To ensure balanced model training and evaluation, a stratified random sampling approach was employed, selecting 85 infected and 90 healthy tobacco plant samples for the training set, while the remaining samples were allocated to the test set. The optimal model for TSWV-infected tobacco plants was determined by evaluating performance criteria. Model fitting and performance evaluation were conducted on a computational platform running Microsoft Windows 10 (64-bit), equipped with an AMD Ryzen 5 4500U processor (with Radeon Graphics) and 16.00 GB of RAM. The main steps of this study are shown in the flow chart (**Figure 5**). The datasets are available in the GitHub repository (https://github.com/smith22357/hyperspectral-dataset: tobacco TSWV hyperspectral-dataset).

### 2.3 Sensitive feature variable selection

Feature selection serves as an effective approach to mitigate the challenges of high dimensionality and strong multicollinearity in hyperspectral data while enhancing model training efficiency (Duan et al., 2019). However, single-feature selection methods exhibit significant limitations in complex disease scenarios due to their inability to comprehensively address feature redundancy, nonlinear interactions, and environmental noise interference (Rasti et al., 2018). To systematically capture the spectral differences driven by the diverse physiological and structural changes (such as necrotic spots, leaf curling, and wilting) in tobacco plants following TSWV infection, we concurrently applied multiple feature extraction methods with distinct strategies.

#### 2.3.1 Traditional statistical methods for feature extraction

Traditional statistical methods, such as the ratio method and correlation analysis, for feature extraction, eliminate the need for assumptions about data distributions or reliance on complex model training. Instead, they directly leverage statistical relationships to extract information, characterized by intuitiveness, rapid implementation, and strong interpretability. Reflectance ratios can enhance spectral feature differences, enabling the rapid identification of spectral bands with significant variations across different samples (Lu et al., 2018). The fundamental principle is that a larger absolute reflectance ratio between two conditions (e.g., healthy vs. diseased) at a specific wavelength corresponds to a greater power to distinguish between them. A practical application of this method is found in the work of Gazala et al. (2013), who used spectral ratios on satellite imagery to identify 688 nm, 750 nm, and 445 nm as key wavelengths for detecting yellow mosaic disease in soybeans. Correlation analysis clarifies the trend variations between spectral bands and sample characteristics, thereby screening out key bands (Eches et al., 2011). Previous research has corroborated the existence of a significant correlation between characteristic wavelengths and disease status in TSWV-infected tomato plants, supporting the validity of this approach (Zhou et al., 2022). Therefore, in this study, the Pearson correlation coefficient between reflectance at each wavelength and the plant health categories is employed to quantify the linear relationship between individual wavelengths and TSWV infection status, thus identifying sensitive features for model development.

#### 2.3.2 Relevant features (relief) for feature extraction

Relief is a typical filtering feature selection method that measures the importance of each feature through correlation statistics and assigns different weight values. It selects k nearest neighbor sample points to deal with incomplete and noisy data, making the feature selector more robust (Urbanowicz et al., 2018). This capability allows Relief to effectively identify key wavelength bands that differentiate locally similar tobacco plants, which may be uniquely sensitive to capturing subtle characteristics such as localized necrosis from TSWV infection. The combination of CNN and Relief methods based on thermal infrared spectroscopy has demonstrated promising results in the classification of tobacco mosaic virus in tobacco plants (Singh et al., 2025).

#### 2.3.3 Recursive feature elimination for feature extraction

Recursive feature elimination (RFE) is a method used to screen for the best combination of features, and the characteristic of the RFE method is to identify environmental covariant quantum sets that significantly contribute to the target variable, avoiding redundancy in the variational quantum sets (He et al., 2021). The algorithm’s objective is to maintain or even improve classification accuracy throughout this process. By iteratively assessing the synergistic discriminative power of feature subsets, RFE effectively reverse-engineers an optimal combination. This makes it well-suited for complex classification problems, such as interpreting the varied symptoms of TSWV-infected tobacco plants. The practical utility of this approach was demonstrated by its integration with logistic regression to identify multiple tomato leaf spot diseases, including TSWV (Ruszczak et al., 2024).

#### 2.3.4 Successive projections algorithm for feature extraction

The SPA algorithm uses projection operations in vector space to obtain a subset of variables with the minimum collinearity. The selected new variable is the maximum projection variable of the unselected variable on the orthogonal subspace of the selected variables, effectively eliminating redundant band information (Soares et al., 2013). By reducing interference, SPA focuses on the most representative and mutually independent spectral features. This results in a set of key indicators where each band corresponds to a distinct physiological dimension affected by TSWV. Consequently, SPA has demonstrated strong performance in extracting feature indicators for early-stage TSWV infection (Gu et al., 2019).

#### 2.3.5 Principal component analysis for feature extraction

PCA is a statistical procedure that utilizes an orthogonal transformation to convert a set of possibly correlated variables into a set of linearly uncorrelated variables called principal components (PCs). PCA can effectively reduce data dimensionality by concentrating most of the original information into a few leading PCs, significantly minimizing redundancy and computational load while preserving essential data structures (Maćkiewicz and Ratajczak, 1993).

#### 2.3.6 Vegetation indices for feature extraction

Unlike the original spectrum, the vegetation index can effectively integrate relevant spectral signals and highlight the spectral characteristics of observation targets (Xue and Su, 2017). Given the symptom characteristics of TSWV (Srinivasan et al., 2017), this study selected VIs that are sensitive to crop growth and crop diseases, such as photosynthesis and pigments, as candidate features for model construction (**Table 2**).

**Table 2:** Vegetation indices used in this study.

Vegetation Index	Formula	References
NDVI (Normalized Difference Vegetation Index)	NDVI=(R_800_-R_670_)/(R_800_+R_670_)	(Tucker, 1979)
NDVI850 (Normalized Difference Vegetation Index)	NDVI850=(R_850_-R_651_)/(R_850_+R_651_)	(Lightle et al., 2001)
TVI (Triangle Vegetation Index)	TVI=0.5[120*(R_761_ – R_581_)- 200*(R_651_ – R_581_)]	(Broge and Leblanc, 2001)
Chl_green_ (Chlorophyll green)	Chl_green_=(R_760_/R_800_)/(R_540_/R_560_)	(Gitelson et al., 2003)
SIPI (Structure Insensitive Pigment Index)	SIPI=(R_800_-R_445_)/(R_800_-R_600_)	(Penuelas et al., 1995)
GI (Green Index)	GI=R_677_/R_554_	(Smith et al., 1995)
DSSI2 (Damage Sensitive Spectral Index 2)	DSSI2=(R_747_-R_901_-R_537_-R_572_)/(R_747-_R_901_+R_537_-R_572_)	(Mirik et al., 2006)
TCARI (Transformed Chlorophyll Absorption in Reflectance Index)	TCARI=3×[(R_700_-R_670_)-0.2×(R_700_-R_550_)×(R_700_/R_670_)]	(Haboudane et al., 2002)
MTCI (MERIS Terrestrial Chlorophyll Index)	MTCI=(R_754_-R_709_)/(R_709_-R_681_)	(Dash and Curran, 2004)
PRI (Photochemical Reflectance Index)	PRI=(R_531_-R_570_)/(R_531_+R_570_)	(Peñuelas et al., 1994)
SRI (Senescence Reflectance Index)	SRI=R_515_/R_550_	(Delegido et al., 2008)

### 2.4 Modeling techniques and performance

Machine learning methods can effectively mine the relationship between spectral characteristics and disease occurrence, which is an important method for tobacco disease monitoring and modeling (Chen et al., 2023). In this study, three machine learning algorithms with strong generalization capabilities—support vector machine (SVM), k-nearest neighbors (KNN), and extreme gradient boosting (XGBoost) —were employed to construct the classification model. To ensure robust model evaluation, a 5-fold cross-validation approach with 5 repeated iterations was implemented. All data analyses and computational modeling were conducted using Python 3.5.

#### 2.4.1 Support vector machine

**Figure 6:**
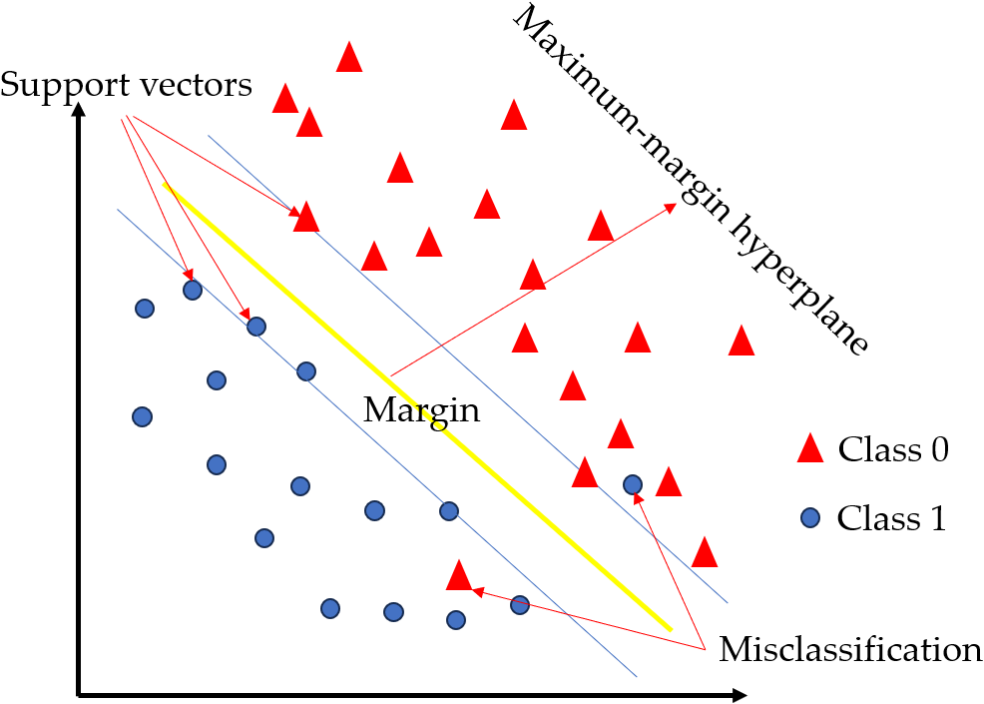
Schematic of SVM maximum-margin classification.

The support vector machine (SVM) algorithm, developed by Cortes and Vapnik (Cortes and Vapnik, 1995), employs a core mechanism illustrated in **Figure 6** (Chauhan et al., 2018): a maximum-margin hyperplane (central yellow line) separates two classes (blue circles/red triangle) using support vectors (boundary points) to define the optimal decision boundary, while allowing misclassifications near the margins to handle noisy data. SVM is a commonly used supervised learning method that can effectively solve linear binary classification problems. By introducing a kernel-based SVM, the vector is projected onto a higher-dimensional space, where the sample vector is linearly separable. In this new model, the maximum-margin hyperplane space is estimated to improve the linear separability of the data (Zhang et al., 2014). A radial basis function (RBF) kernel was used for the SVM model. The optimal parameters (cost and gamma) were determined by evaluating different combinations and selecting the settings that yielded the highest validation accuracy. Using this approach with texture features, the SVM model achieved the highest classification accuracy of 99.83% for identifying TSWV (Mokhtar et al., 2015).

#### 2.4.2 K-nearest neighbor

**Figure 7:**
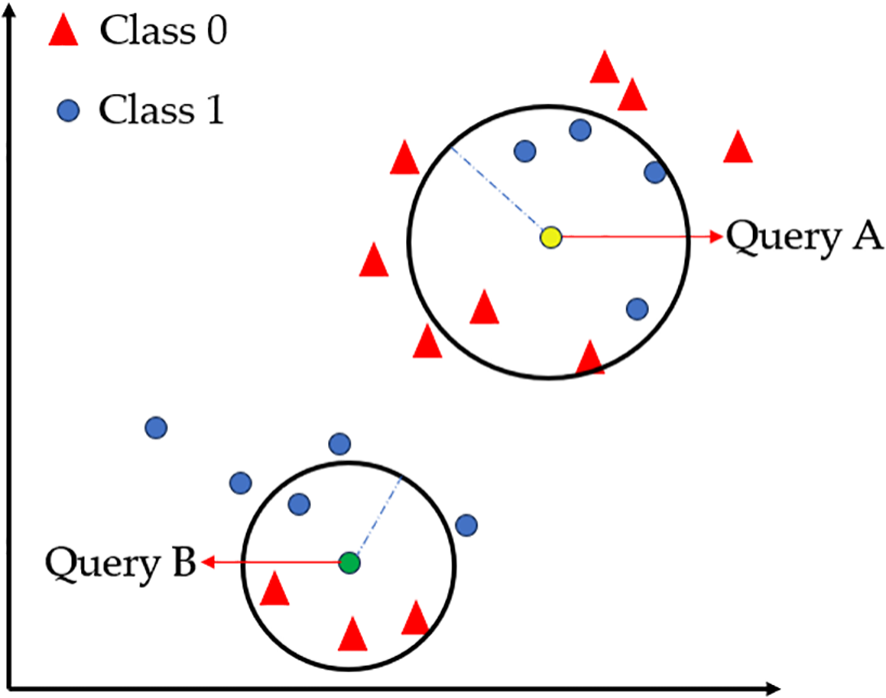
KNN classification: Visualization of neighborhood density and spatial distribution.

The k-nearest neighbor (KNN) algorithm was first proposed by Fix and Hodges (Fix, 1985). The principle of the KNN classifier is to find the nearest training sample for the test sample through distance measurement, and decide the class of the test sample based on the class of the training sample. The KNN algorithm is based on this principle. Given the class of training sample labels, it searches for the k most similar or nearest training samples of the test sample, and then decides the class of the test sample based on the k nearest training sample classes. As shown in **Figure 7** (Uddin et al., 2022), two clustering regions (Class 0/Class 1) are represented by red triangles and blue circles, respectively. When the query point Query A is positioned near the Class 1 cluster, it is classified as Class 1 (determined by the density of neighboring points), while Query B follows the same principle. This intuitively demonstrates KNN’s core mechanism based on local majority voting (Deng et al., 2016). The practical efficacy of this algorithm is highlighted by a model combining InceptionResNetV2 and KNN, which achieved 99.00% accuracy in classifying okra plants infected with yellow vein mosaic virus (Mittal et al., 2024).

#### 2.4.3 Extreme gradient boosting

**Figure 8:**
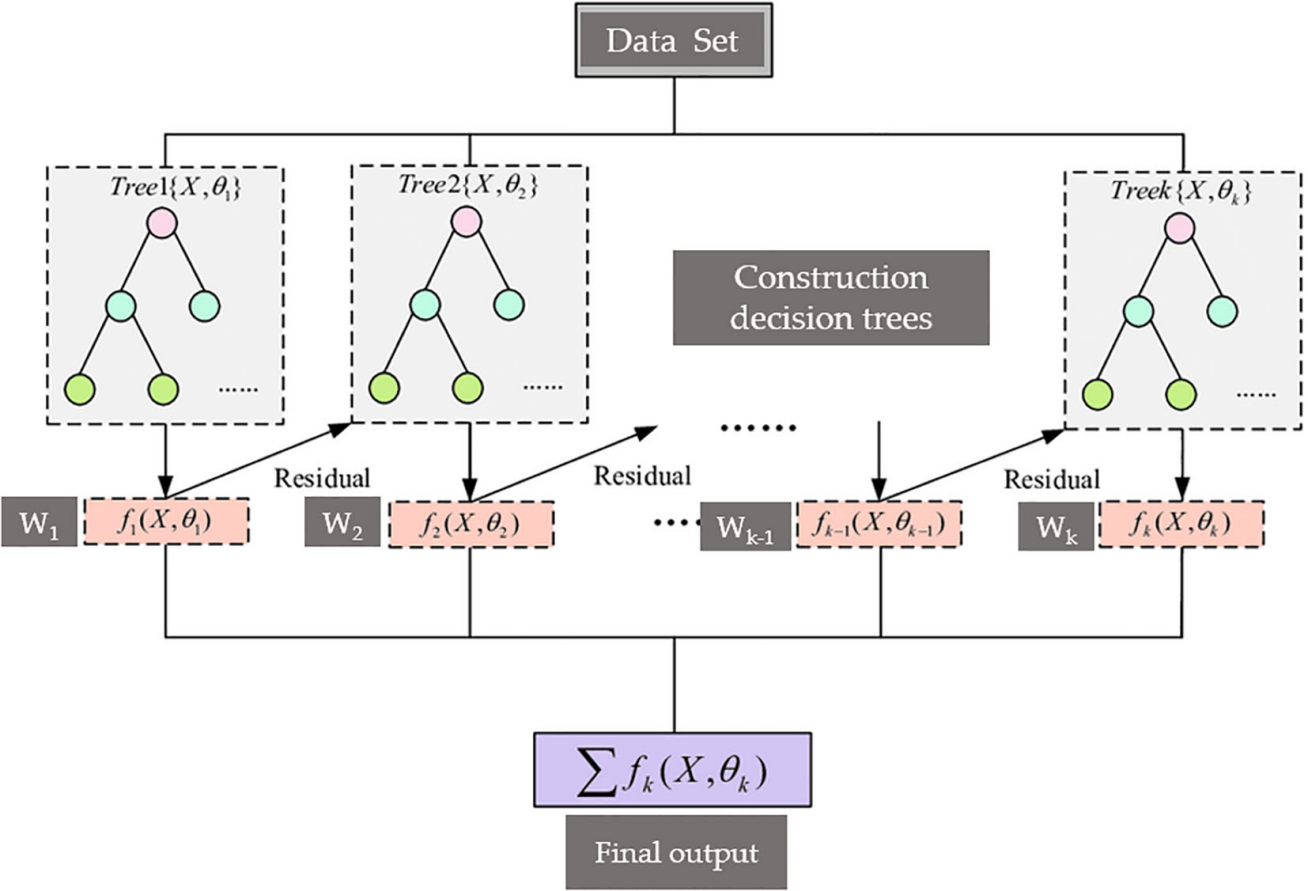
Workflow of XGBoost: Residual learning and weighted ensemble.

The extreme gradient boosting (XGBoost) algorithm, proposed by Chen and Guestrin, belongs to the boosting algorithm (Chen and Guestrin, 2016), which combines numerous weak classifiers to form a strong classifier. It applies the idea of an ensemble tree and optimizes based on GBDT, using a second-order Taylor expansion to accelerate gradient descent and introducing a regularization penalty term to solve the optimal objective function. It has the advantages of parallel optimization, custom loss, and prevention of overfitting. **Figure 8** (Khan et al., 2022) illustrates the algorithm core workflow: Starting from the original Data Set, weighted data sampling feeds parallel decision trees that iteratively process residual errors to generate prediction outputs (W_k_), which are summed via summation to produce the Final output – visually capturing its additive ensemble mechanism and gradient-boosted residual learning. In application, a model integrating VGG with XGBoost was used to classify nearly 10 different tomato leaf diseases, such as TSWV (Aravind et al., 2025).

#### 2.4.4 Model evaluation

This study employed overall accuracy (OA), Kappa coefficient, area under the receiver operating curve (AUC) to evaluate model performance. The Kappa statistic quantifies the agreement between model predictions and true labels after explicitly correcting for the agreement expected by random chance. Unlike OA, which may provide an inflated estimate of performance under class imbalance or skewed prevalence, Kappa isolates the chance-corrected portion of agreement and therefore offers a more rigorous and interpretable measure of true classification reliability. A higher Kappa value indicates that the model’s improvement over random guessing is quantitatively substantial. The AUC measures the model’s discriminative ability across all possible decision thresholds, rather than at a single operating point as in OA. Specifically, AUC represents the probability that a randomly selected positive sample will receive a higher predicted score than a randomly selected negative sample. This threshold-independent nature allows AUC to capture the model’s overall ranking capability and robustness, particularly in situations where OA may be insensitive or misleading. An AUC value approaching 1 indicates strong separation between the two classes and a high probability of correct ordering. To establish a robust and reliable TSWV recognition model, the 5-fold cross-validation method was utilized to reduce the differences between models.

## 3 Results

### 3.1 Binary classification of vegetation and non-vegetation

**Figure 9:**
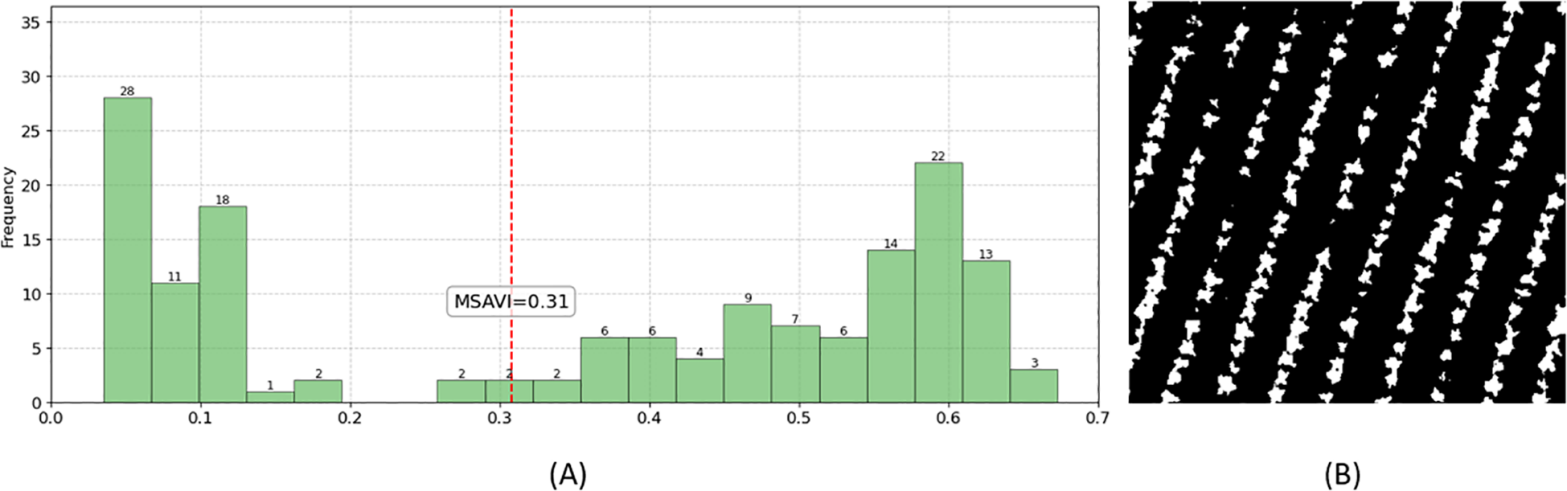
Background mask generated by the MSAVI-Otsu method. **(A)** Threshold determination using MSAVI-Otsu; **(B)** Resulting background mask image.

As shown in **Figure 9A**, the MSAVI-Otsu method automatically determined the optimal threshold (MSAVI = 0.31) to distinguish tobacco plants from non-vegetation backgrounds based on the MSAVI distribution (vegetation: MSAVI ≥ 0.31; non-vegetation: MSAVI < 0.31). When applied to the orthomosaic, this threshold produced a binary mask (**Figure 9B**), where non-vegetation pixels were uniformly classified as background, isolating tobacco plants as contiguous white regions.

### 3.2 Spectral responses to healthy and TSWV-infected tobacco plants

**Figure 10:**
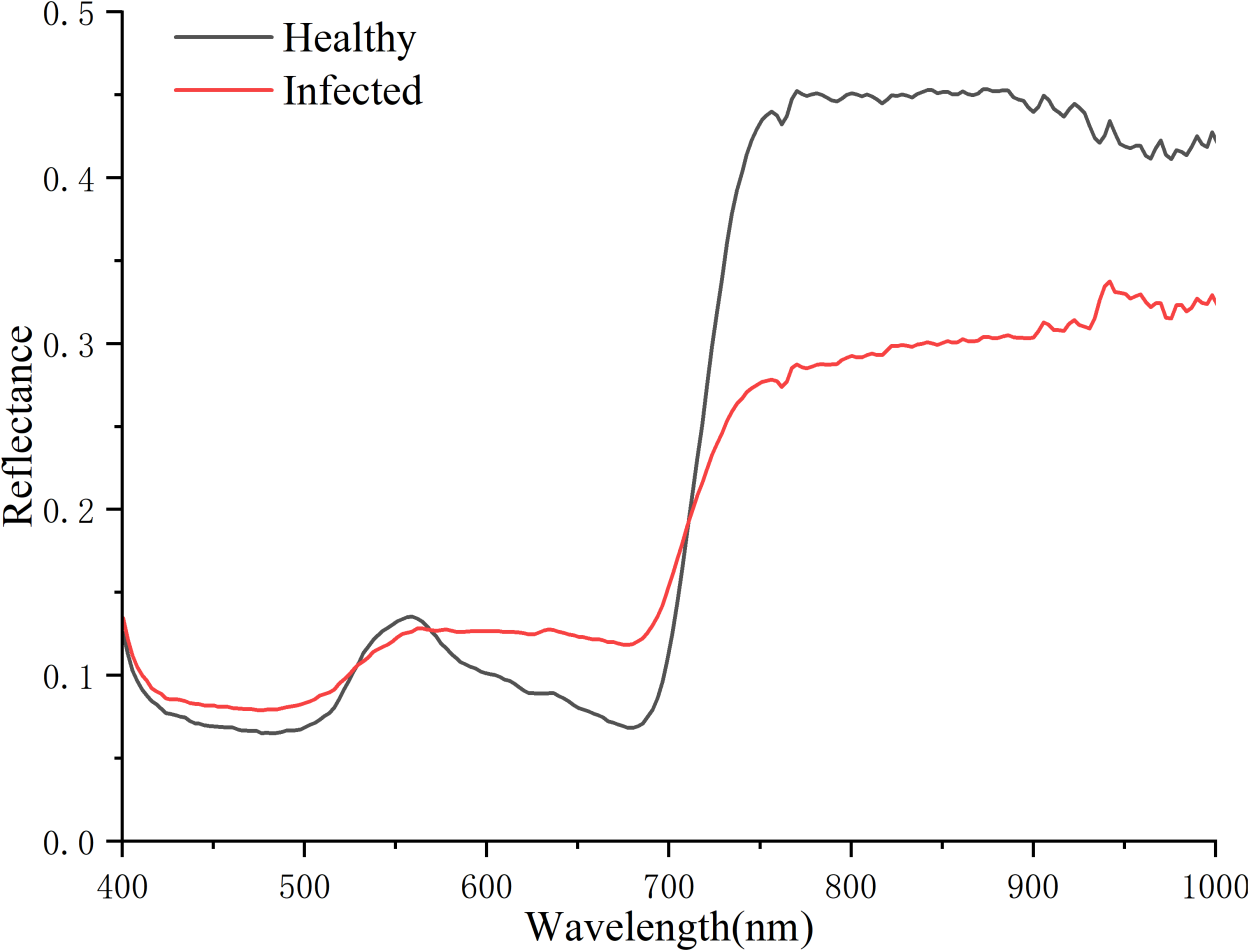
Spectral responses to healthy and infected tobacco plants.

As shown in **Figure 10**, healthy and infected tobacco plants exhibit distinct differences in the red and near-infrared regions. In the red region, both healthy and infected plants show an absorption valley near 680 nm, but the overall reflectance of infected plants is higher than that of healthy plants. In contrast, the reflectance of infected plants in the near-infrared region is significantly lower than that of healthy plants. These findings indicate that the red and near-infrared regions are critical for distinguishing between healthy and infected tobacco plants, and are hence identified as key areas for selecting spectral feature indicators.

### 3.3 Feature extraction methods for spectral indicator

**Figure 11:**
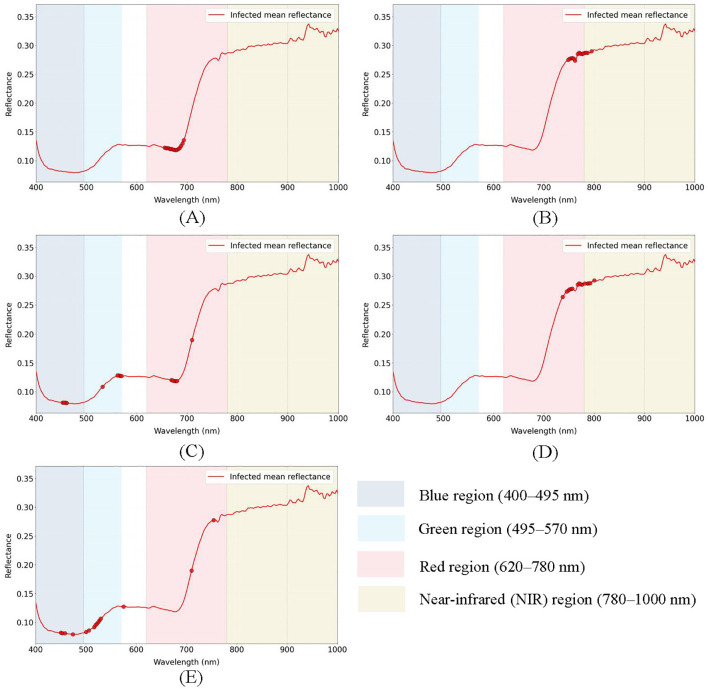
Extracting features by different methods. **(A)** Ratio, **(B)** Correlation, **(C)** PCA, **(D)** Relief, **(E)** SPA.

**Figure 11A** presents the reflectance analysis results based on the ratio methodology, demonstrating marked discrepancies in the mean reflectance ratios between healthy and infected tobacco plants. Compared to the original reflectance spectra, the ratio method substantially enhanced spectral feature discrimination. The top 15 bands with the largest absolute ratio values were selected as feature indicators, primarily concentrated in the red-light region of 650–690 nm, with the maximum ratio of 1.75 observed at 682.8 nm.

**Figure 11B** illustrates the correlation analysis between spectral reflectance and disease progression in tobacco plants, where healthy and infected samples were numerically encoded as 0 and 1, respectively. Bands with higher absolute correlation coefficients possess greater discriminative value for sample differentiation. The 15 bands with the largest absolute correlation coefficients were selected as feature indicators, predominantly concentrated within the 740–790 nm range, corresponding to the red-edge and near-infrared regions.

**Figure 11C** displays the characteristic bands selected after dimensionality reduction of the spectral data using Principal Component Analysis (PCA). PCA effectively reduces spectral dimensionality by determining the number of principal components based on a cumulative variance contribution rate exceeding 95%, thereby identifying key spectral bands. The results indicated that the first three principal components accounted for 96.30% of the cumulative variance. Consequently, the top 5 loading bands from each of these three principal components (totaling 15 bands) were selected as feature indicators. These characteristic bands were distributed across the blue, green, and red-light regions.

**Figure 11D** demonstrates the feature bands selected by the Relief algorithm, which evaluates the contribution of each band to class discrimination through weight assignment, where higher weights indicate greater importance for classification. The top 15 bands with the highest weights were selected as feature indicators, mainly concentrated in the 740–790 nm range (red-edge and near-infrared regions). These results show considerable similarity with those obtained from correlation analysis.

**Figure 11E** presents the characteristic bands selected by the Successive Projections Algorithm (SPA), which minimizes inter-band information redundancy to identify features with the lowest collinearity. The results show that 15 bands with the lowest collinearity were selected as feature indicators, distributed across blue (450–480 nm), green (500–580 nm), and red-light regions (710–760 nm), with particular concentration in the blue and green regions.

### 3.4 Identification of healthy and infected tobacco plants

Three machine learning algorithms (SVM, KNN, XGBoost) were used to classify the healthy and infected tobacco samples with sensitive feature variables by Ratio, PCA, Correlation, Relief, SPA and VIs, respectively.

#### 3.4.1 Determination of model parameters

**Table 3**, **Figure 12** show the model performances based on different combinations of parameters as well as the optimal parameter settings. This study employed 5 repetitions of 5-fold cross-validation to evaluate the overall classification accuracy (%) for each parameter set, with the optimal parameters determined by maximizing the mean classification accuracy. The results demonstrate that parameter optimization has a decisive impact on machine learning model performance. The lower coefficient of variation (CV) for overall accuracy (OA) revealed the stability of the KNN and XGBoost models under different parameter settings among the three machine learning algorithms. The CV for SVM-based combinations ranged between 0.37% and 15.28%, while those for KNN-based combinations fell within 0.15%–0.50%, and XGBoost-based combinations ranged from 0.00% to 0.87%. Overall, the CV of SVM-based combinations exhibited greater fluctuation, whereas the CV values of KNN-based and XGBoost-based combinations remained relatively stable. Among all algorithm configurations, the combinations with the lowest CV were SVM-Correlation, KNN+VIs, and XGBoost+Ratio. The CV values for the combinations of SVM and KNN with Relief were at an intermediate level, recorded at 0.80% and 0.24%, respectively.

**Table 3:** Descriptive statistics of the CV (%) of cross-validation over 5 iterations for each combination of parameters.

Model	Sensitive features	Optimal parameters	CV (%)
SVM	Ratio	C: 10, gamma: 0.01	1.57
	Correlation	C: 1, gamma: 0.01	0.37
	PCA	C: 10, gamma: 0.01	13.94
	Relief	C: 100, gamma: 0.1	0.80
	SPA	C: 10, gamma: 0.01	15.28
	VIs	C: 10, gamma: 0.1	0.83
KNN	Ratio	n_neighbors: 3, weights: uniform	0.50
	Correlation	n_neighbors: 5, weights: uniform	0.48
	PCA	n_neighbors: 7, weights: uniform	0.46
	Relief	n_neighbors:5, weights: uniform	0.24
	SPA	n_neighbors:5, weights: uniform	0.72
	VIs	n_neighbors:5, weights: uniform	0.15
XGBoost	Ratio	learning_rate: 0.01, max_depth: 3	0.00
	Correlation	learning_rate: 0.01, max_depth: 3	0.87
	PCA	learning_rate: 0.01, max_depth: 3	0.28
	Relief	learning_rate: 0.01, max_depth: 3	0.81
	SPA	learning_rate: 0.01, max_depth: 5	0.38
	VIs	learning_rate: 0.01, max_depth: 3	0.65

**Figure 12:**
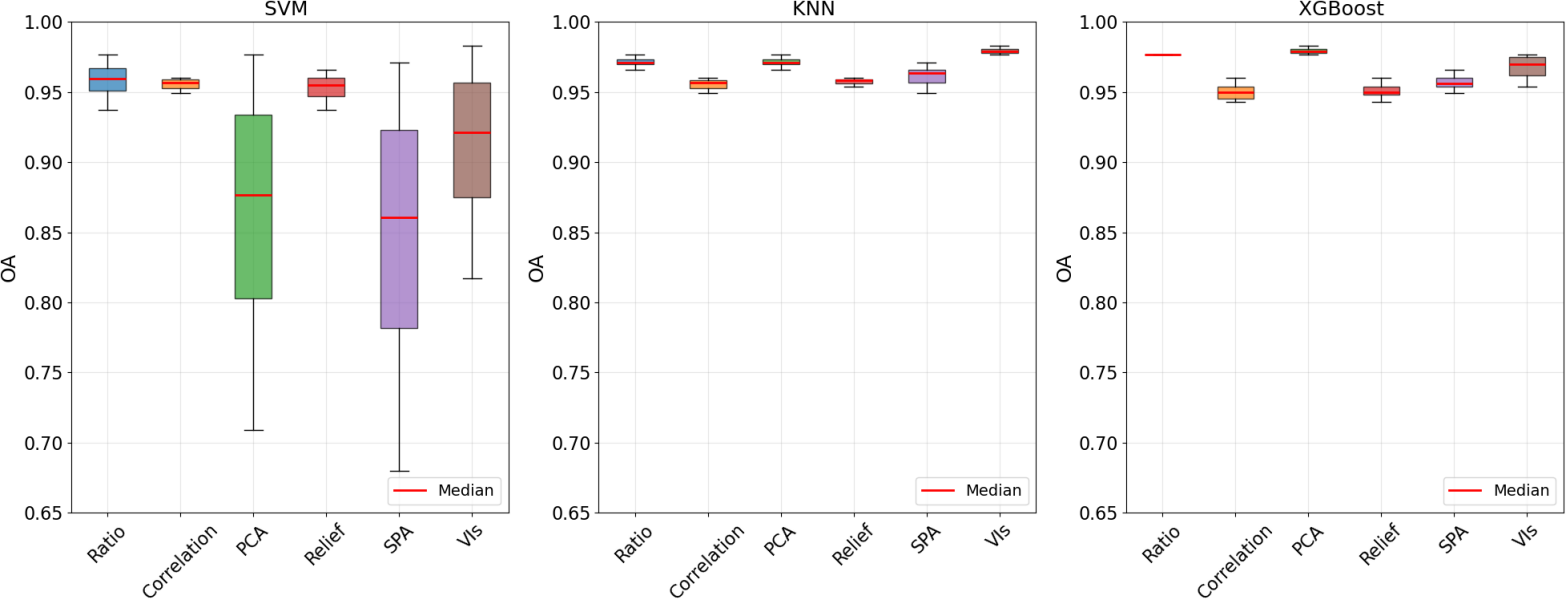
The result of the OA (%) of cross-validation over 5 iterations for each combination of parameters.

**Figure 13:**
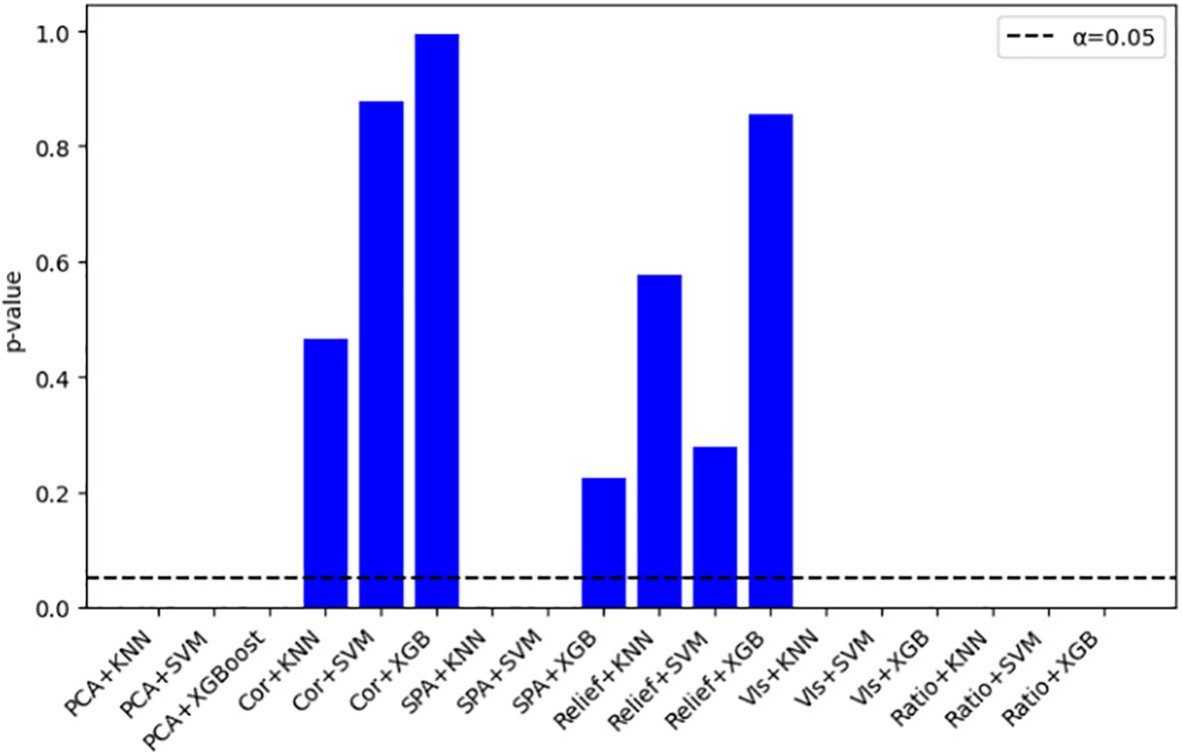
P-value of paired tests for each model.

**Figure 13** displays the paired T-test results of 18 combined models using optimal parameters after 5-fold cross-validation repeated 5 times. The results indicate that the performance differences for the combination models of Correlation and Relief with three algorithms, as well as the SPA-XGBoost model, were not statistically significant. This lack of significant disparity, consistent across repeated cross-validation folds, suggests that these seven models exhibit stable and consistent predictive performance, which is a key characteristic of robust generalization ability (Hamarashid, 2021).

#### 3.4.2 Performance assessment of TSWV identification models

**Figure 14:**
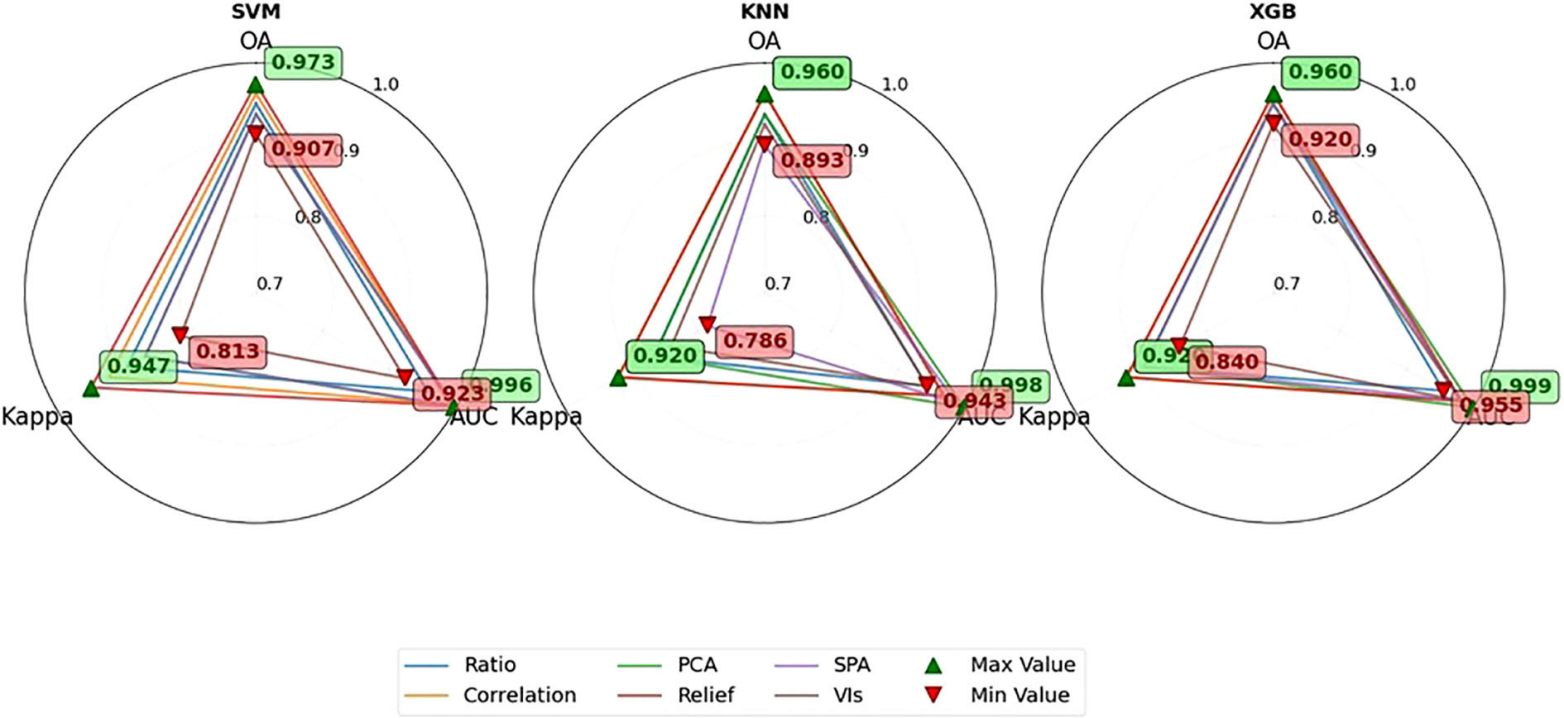
Model performance of three machine learning techniques with six sensitive feature selection methods.

The performance evaluation results of the 18 models constructed based on optimal parameters on the test set are shown in **Figure 14**. The average OA of the different combined models ranged from 89.3% to 97.3%, with AUC values between 0.923 and 0.999, and Kappa coefficients distributed in the interval of 0.786-0.947. Regarding different combinations of sensitive feature variables, models utilizing features selected by Relief and Correlation algorithms demonstrated superior performance, achieving OA between 96.0% and 97.3%. Comparing the model performance of different combinations, the SVM model combined with the Relief method achieved the best performance with an OA of 97.3%, followed by the SVM model combined with the Correlation method with an OA of 96.0%. A comprehensive analysis indicates that the SVM-Relief and SVM-Correlation combinations not only exhibited lower CV on the training set but also demonstrated higher OA values on the test set, and both showed no statistically significant differences in the paired t-test, highlighting their outstanding overall performance.

Based on the best-performing SVM-Relief combination, to further reduce the feature dimensionality and improve model performance, a composite feature extraction strategy (termed RPR) was adopted. The approach first applied PCA to reduce the dimensionality of the features extracted by Relief. Then, RFE based on SVM was used to iteratively remove features according to their importance and select the feature subset with the OA, which was subsequently evaluated on the test set. PCA results indicated that the first principal component (PC1) accounted for 99.8% of the cumulative variance contribution. The top six spectral indicators with the highest loadings in PC1 (775.6/772.9/781.1/756.4/753.7/750.9 nm) were selected as input features for RFE. As shown in **Table 4**, when the number of features was gradually reduced from six to three, the OA on the training set remained consistently at 96.6%. When the feature set was reduced to four indicators (775.6/772.9/781.1/756.4 nm), the model achieved optimal performance on the test set (OA = 97.3%, Kappa = 0.947, AUC = 0.990), which was comparable to the performance obtained using all 15 original features.

**Table 4:** The impact of RPR feature selection on model effectiveness.

Sensitive features	Optimal parameters	Training data	Test data		
		OA	OA	AUC	Kappa
775.6nm, 772.9nm, 781.1nm, 756.4nm, 753.7nm, 750.9nm	C: 0.1, gamma: 0.01 kernel: rbf	0.966	0.960	0.992	0.920
775.6nm, 772.9nm, 781.1nm, 756.4nm, 753.7nm	C: 0.1, gamma: 0.01 kernel: rbf	0.966	0.960	0.990	0.920
775.6nm, 772.9nm, 781.1nm, 756.4nm	C: 0.1, gamma: 1 kernel: poly	0.966	0.973	0.990	0.947
775.6nm, 772.9nm, 781.1nm	C: 0.1, gamma: 1 kernel: poly	0.966	0.960	0.981	0.920

Therefore, the feature combination selected by the SVM-RPR method (775.6/772.9/781.1/756.4 nm) is suitable for distinguishing between healthy and TSWV-infected tobacco plants based on hyperspectral imagery, as it maintains model performance while significantly reducing feature dimensionality.

## 4 Discussion

The findings demonstrated the feasibility of UAV-based hyperspectral imaging combined with machine learning for field-scale identification of TSWV-infected tobacco plants. Among the evaluated combinations of feature selection algorithms and classifiers, the SVM-RPR model achieved superior classification accuracy, underscoring its efficacy in distinguishing TSWV-infected from healthy plants under complex field conditions.

### 4.1 Spectral response analysis of TSWV-infected tobacco plants

The invasion of pathogens into plant leaves leads to the occurrence of their physiological characteristics, which is the basis for remote sensing to identify healthy and diseased plants (Zarco-Tejada et al., 2013). This study compared and analyzed the hyperspectral reflectance of healthy and infected tobacco plants and found that there were significant differences in spectral reflectance between healthy and infected tobacco plants in the visible red-light region, red-edge region, and NIR region. Similar spectral feature differences were also found in the identification of tomato bacterial spot disease (Abdulridha et al., 2020) and wheat scab (Bauriegel et al., 2011).

Red-edge represents the region of abrupt change in leaf reflectance between 680 and 780 nm caused by the combined effects of strong chlorophyll absorption in the red wavelengths, and high reflectance in the NIR wavelengths due to leaf internal scattering (Cho and Skidmore, 2006). As a distinctive spectral feature of green vegetation, the red edge is closely associated with crop chlorophyll content (Gupta et al., 2003). When disease infection leads to crop tissue necrosis or withering, healthy tissues exhibit significantly higher reflectance in the red-edge band compared to infected tissues (Scholes and Rolfe, 1996), making this spectral region particularly valuable for detecting symptomatic diseases. Our study reveals that the features (775.6/772.9/781.1/756.4 nm) in the best-performing model fall within the red-edge region. Consistency was observed with the demonstration that the wavelength of 697.17 nm in the red-edge region is closely related to TSWV infection in tobacco leaves (Krezhova et al., 2012). However, the effective bands identified in our field-scale study (775.6/772.9/781.1/756.4 nm) differ from the 828–856 nm bands reported in previous leaf-scale research (Gu et al., 2019). This discrepancy likely arises from the dilution effect of canopy-level mixed pixels on individual leaf spectral features (Zhao et al., 2022), resulting in a shifted sensitivity toward the red-edge region. Simultaneously, previous research has predominantly targeted pre-necrotic TSWV detection in tobacco leaves, focusing on spectral alterations prior to visible symptom formation (Gu et al., 2019), whereas our study centers on identifying diseased plants after lesion emergence. This fundamental distinction in the disease progression stage examined may explain the divergence between the field-scale spectral signatures we identified and leaf-scale features reported in prior literature, constituting a principal methodological distinction of this work.

This study employed a UAV-based hyperspectral imaging system (400–1000 nm) to identify sensitive spectral bands for TSWV detection in tobacco plants during the rosette stage. To ensure the accuracy and reliability of TSWV-infected plant detection while minimizing interference from other biotic or abiotic factors on TSWV-sensitive spectral features, standardized cultivation practices with uniform irrigation and fertilization were adopted. This approach effectively eliminated significant nutrient deficiencies (particularly nitrogen stress) during this growth phase, thereby eliminating the influence of water and nutrient stress on red-edge bands. Additionally, the low canopy coverage of rapidly growing rosette-stage tobacco minimized the occurrence of fungal or bacterial leaf spot diseases. Although migratory pests drive high transmission rates of TSWV and TMV during this period, the two diseases exhibit distinct pathogenic mechanisms: TMV is characterized by intracellular inclusion bodies, with its diagnostic spectral bands at 639.04, 697.44, 719.15, 749.90, 874.91, 938.22, and 971.78 nm (Zhu et al., 2017), whereas TSWV induces large-scale chlorophyll degradation and necrotic spotting, leading to unique spectral signatures in the red-edge region (e.g., 775.6/772.9/781.1/756.4 nm). This distinction provides a plausible explanation for detecting TSWV-infected tobacco plants using red-edge characteristic bands.

### 4.2 Advantages of feature extraction methods

The impact of diseases on plant bio-physical parameters (including chlorophyll, water content, and cellular structure) manifests as continuous or near-continuous alterations in reflectance spectra. Given the inherent high correlation between adjacent bands in hyperspectral imagery, the spectral sensitivity to a particular disease class is rarely isolated to a single band. Instead, it is typically exhibited across a range of neighboring wavelengths (Sun and Du, 2019). This characteristic underscores the importance of employing effective feature extraction methods to accurately identify disease-sensitive spectral bands while suppressing noise interference, which is essential for constructing robust disease identification models. This study demonstrates that models developed using correlation analysis and the Relief feature selection method achieve notably strong performance, which is closely tied to their distinct operational mechanisms. Correlation analysis adopts a global perspective, calculating the correlation between each spectral band and the class labels, thereby prioritizing the selection of bands highly correlated with the labels as features (Hall, 1999). In contrast, Relief operates from a local perspective by identifying nearest neighbors for each sample and comparing feature differences between these neighbor pairs. A feature receives a high score if it exhibits substantial differences in neighbor pairs of different classes but minimal differences in neighbor pairs of the same class (Urbanowicz et al., 2018). Since disease-induced spectral signals are continuous and jointly carried by adjacent bands, both correlation analysis—based on “global correlation”—and Relief—based on “local discriminative capability”—prove effective in identifying characteristic wavelengths sensitive to diseases. These findings align with previous studies (Bhatia et al., 2020; Takkar et al., 2021), which also reported that feature extraction methods based on correlation and Relief consistently achieve superior performance across multiple plant disease classification tasks.

Additionally, the models utilizing SPA demonstrated inferior performance in this study. The underlying reason is that SPA’s selection paradigm is a spectral variable selection method designed to eliminate redundancy and minimize collinearity, prioritizing the selection of mutually linearly independent bands over those with strong discriminative power (Soares et al., 2013). However, since the diagnostically critical red-edge region is characterized by high band correlation, SPA tends to bypass it, opting for less collinear bands in the blue-green spectrum (Cho and Skidmore, 2006). Consequently, this selection strategy leads to reduced model accuracy, aligning with the results reported by Araújo et al. (2001).

### 4.3 Model effectiveness and generalization ability

Machine learning algorithms exhibit distinct performance characteristics in hyperspectral disease detection tasks. In our comparative analysis of three machine learning techniques, KNN and XGBoost demonstrated lower accuracy coefficients of variation and superior robustness in recognizing TSWV-infected tobacco plants compared to SVM. This can be explicitly explained by the fact that the performance of SVM is highly dependent on the kernel function and its parameter γ, as well as the regularization parameter C. The combination of C and γ creates a complex hyperparameter search space characterized by a sharp performance “peak”. Slight deviations from the optimal parameter set can lead to significant fluctuations in Overall Accuracy (OA), resulting in a higher coefficient of variation. However, once a suitable (C, γ) combination is identified, model performance can be substantially improved (Ali et al., 2023). In contrast, XGBoost excels at capturing high-dimensional feature interactions through gradient-boosted decision trees, which confers inherent robustness, particularly in small-sample scenarios (Chen and Guestrin, 2016). Meanwhile, the performance of the KNN model typically exhibits gradual variation with changes in the parameter K. Since the accuracy does not change abruptly with minor adjustments to K, its coefficient of variation remains relatively low (Guo et al., 2003).

Model performance is closely tied not only to feature relevance but also to the compatibility between feature data structures and machine learning algorithm properties (Kaur et al., 2019). This study demonstrates that the combination of features selected by the Relief algorithm with an SVM classifier yields superior results compared to its combinations with KNN and XGBoost. The essence of the Relief algorithm is highly similar to that of SVM, as both aim to identify and reinforce the “classification boundary” (Urbanowicz et al., 2018). The underlying “boundary discrimination” criterion of Relief’s feature selection process aligns strongly with the SVM classifier’s objective of “maximizing the margin”. The feature subset provided by Relief minimizes the complexity of the feature space and accentuates the classification boundary, enabling the SVM to more effectively identify the generalization-optimal hyperplane, thereby achieving the highest classification accuracy (Dai et al., 2015). This likely explains the superior performance of the SVM-Relief combination. Previous studies have demonstrated that the approach combining SVM with the Relief algorithm has achieved promising results in hyperspectral-based applications such as variety screening (Qi et al., 2011), disease identification (Dai et al., 2015), and vegetation classification (Miftahushudur et al., 2019).

### 4.4 Practical implications and limitations

The successful application of UAV hyperspectral imaging combined with machine learning for TSWV detection in tobacco plants offers significant practical value for precision agriculture. By identifying infected plants, farmers can implement targeted disease management strategies, such as localized pesticide application or removal of infected plants, thereby reducing economic losses and minimizing unnecessary chemical usage. Nevertheless, the scalability of drone-mounted hyperspectral systems remains hindered by three critical limitations: (1) prohibitively high equipment costs, (2) computationally intensive data preprocessing pipelines, and (3) reliance on high-throughput computing infrastructure. To address these challenges, future research should prioritize the development of cost-effective multispectral sensors tailored for *in situ* disease surveillance, leveraging the TSWV diagnostic spectral bands (e.g., 775.6/772.9/781.1/756.4 nm) identified in this study. Further optimization could involve embedding the SVM-RPR classification model into UAV edge computing architectures. Such integration would facilitate real-time generation of georeferenced TSWV distribution heatmaps, empowering growers to execute timely precision interventions. This paradigm shift toward edge-processed, field-deployable solutions may substantially enhance the operational feasibility of aerial phytopathology monitoring in resource-limited agricultural settings. This also aligns with global trends toward sustainable agriculture and reduced environmental impact.

This study focuses specifically on identifying diagnostic spectral bands for TSWV-infected plants after symptom manifestation and develops a machine learning-based disease detection model. To mitigate the influence of complex canopy structures, images were acquired at the rosette stage with minimal shading to avoid mutual occlusion between plants. A 3×3 spatial–spectral unit was adopted for mean-based statistical processing to reduce random noise through spatial averaging and to capture the small-scale patchy patterns associated with disease (Peng et al., 2022). Although these steps reduce the impact of complex canopy structures, they cannot fully eliminate scale effects from plant architecture. Future work will therefore integrate spectral with structural data (e.g., LiDAR, point clouds) for plant-level segmentation and correction. Incorporating multi-angle and multi-temporal observations will further capture symptom evolution for more robust early detection. In addition, compared to the millimeter-scale resolution required for early detection, the 5 cm ground sampling distance (GSD) hyperspectral imagery used in this study poses significant challenges for identifying early-stage TSWV infections. Although threshold-based methods effectively eliminate background interference (e.g., soil, plastic mulch), the manually delineated 3×3 pixel ROI-averaged spectra still fail to target small lesion areas during early infection. To overcome these limitations, future work will leverage hyperspectral-multispectral image fusion to enhance spatial resolution and integrate deep learning for automated lesion detection and disease severity grading.

This study successfully developed a classification model for healthy and TSWV-infected tobacco plants using UAV-based hyperspectral imaging. However, as the model is a binary classifier for health status, it cannot yet resolve or quantify specific TSWV symptom features. Furthermore, since the infected samples were collected from naturally infected field populations—and although labeling was based on typical TSWV symptoms while excluding signs of other viruses—symptom-based screening still entails a degree of uncertainty. Future work will employ artificial inoculation to ensure samples are infected specifically with TSWV, and will incorporate object detection or image segmentation algorithms to achieve symptom localization and quantification.

## 5 Conclusions

This study utilized a UAV hyperspectral camera (400–1000 nm) to capture field-scale imagery of tobacco plants at the rosette stage. Six feature extraction methods were integrated with three machine learning algorithms (SVM, KNN, XGBoost) to construct 18 identification models. Through systematic comparison of the 18 models’ performance, it was found that the SVM-Relief combination achieved optimal performance, with an overall accuracy of 97.3% (AUC = 0.994, Kappa=0.947) for identifying TSWV in tobacco plants, followed closely by the SVM-Correlation model at 96.0% accuracy (AUC = 0.991, Kappa=0.920). The feature indicators extracted by Relief and Correlation were very similar, with 15 feature bands mostly concentrated in the 740–780 nm range. These results suggest that the red edge region of 740–780 nm holds significant value for distinguishing between healthy and TSWV-infected tobacco plants. Additionally, the combination of SVM and Relief was found to be more suitable for identifying TSWV-infected tobacco plants compared to other combinations.

Furthermore, the proposed SVM-RPR model derived from the combination of PCA and RFE with SVM achieved comparable performance (OA = 97.3%, AUC = 0.990, Kappa = 0.947) to the SVM-Relief model while reducing the number of feature indicators from 15 to 4 (775.6/772.9/781.1/756.4 nm). This finding not only further optimizes the SVM-Relief model but also reconstructs a feature selection method combining Relief-PCA-RFE (RPR), providing a new approach for other hyperspectral-based plant disease identification studies.

The study offers a novel methodology for large-scale monitoring of TSWV incidence in tobacco, while providing critical technical insights for managing this devastating disease across agricultural systems. By enabling detection and targeted interventions, this approach holds significant potential for reducing pesticide use, mitigating crop losses, and advancing sustainable agricultural practices.

## Data Availability

The raw data supporting the conclusions of this article will be made available by the authors, without undue reservation.
